# Molecular and Cellular Substrates for the Friedreich Ataxia. Significance of Contactin Expression and of Antioxidant Administration

**DOI:** 10.3390/molecules25184085

**Published:** 2020-09-07

**Authors:** Antonella Bizzoca, Martina Caracciolo, Patrizia Corsi, Thea Magrone, Emilio Jirillo, Gianfranco Gennarini

**Affiliations:** Department of Basic Medical Sciences, Neurosciences and Sensory Organs, Medical School, University of Bari, Piazza Giulio Cesare, 11. I-70124 Bari, Italy; antonella.bizzoca@uniba.it (A.B.); martina.p.caracciolo@gmail.com (M.C.); patrizia.corsi@uniba.it (P.C.); thea.magrone@uniba.it (T.M.); emilio.jirillo@uniba.it (E.J.)

**Keywords:** Friedreich Ataxia, neurodegeneration, nervous tissue repair, neural cells interactions, transmembrane signaling, polyphenols

## Abstract

In this study, the neural phenotype is explored in rodent models of the spinocerebellar disorder known as the Friedreich Ataxia (FA), which results from mutations within the gene encoding the Frataxin mitochondrial protein. For this, the M12 line, bearing a targeted mutation, which disrupts the Frataxin gene exon 4 was used, together with the M02 line, which, in addition, is hemizygous for the human Frataxin gene mutation (Pook transgene), implying the occurrence of 82–190 GAA repeats within its first intron. The mutant mice phenotype was compared to the one of wild type littermates in regions undergoing differential profiles of neurogenesis, including the cerebellar cortex and the spinal cord by using neuronal (β-tubulin) and glial (Glial Fibrillary Acidic Protein) markers as well as the Contactin 1 axonal glycoprotein, involved in neurite growth control. Morphological/morphometric analyses revealed that while in Frataxin mutant mice the neuronal phenotype was significantly counteracted, a glial upregulation occurred at the same time. Furthermore, Contactin 1 downregulation suggested that changes in the underlying gene contributed to the disorder pathogenesis. Therefore, the FA phenotype implies an alteration of the developmental profile of neuronal and glial precursors. Finally, epigallocatechin gallate polyphenol administration counteracted the disorder, indicating protective effects of antioxidant administration.

## 1. Introduction

The Friedreich Ataxia (FA) is an autosomal neurodegenerative disorder, which affects the development of the long-range axonal trajectories, which grow along the spinal cord and include both sensory and spino-cerebellar afferents [1,2,3]. The corresponding tissue alteration affects sensory as well as spino-cerebellar functions [2,4,5,6,7,8,9,10,11,12,13] and, as such, it results in an ataxic phenotype [9,14,15,16]. Due to concomitant myocardiocyte damage, neurological symptoms are associated with a cardiovascular phenotype [4,5,17,18,19], resulting in ventricular failure. The neural and the cardiovascular phenotypes share a common pathogenic mechanism, based on the evidence that FA is a mitochondrial disorder, affecting at the same time the nervous and the hearth tissue [6,12,20,21,22,23] as a consequence of the mutation of the gene encoding the mitochondrial protein Frataxin [24]. This protein plays a relevant role in modulating oxidative phosphorylation, consistent with the contribution of the mutation of the underlying gene to the disorder pathogenesis, in turn implying mitochondrial iron and sulfur accumulation within the nervous and the cardiac tissues, which results in their degeneration [7,12,23,25,26,27,28,29,30,31,32].

As for their derivation at the tissue level, the perikarya of most of the neurons affected by the Frataxin gene mutation map within the dorsal root ganglia as well as within the cerebellar dentate gyrus, which therefore represents the primary targets of the disorder [33]. At the cellular level, Frataxin gene mutation results in high sensitivity of the affected tissues to oxidative stress [7,12,34], which, in turn, is also relevant for devising potential therapeutic approaches, based on the administration of antioxidants components, demonstrated to be helpful also in other neurodegenerative disorders as shown for Idebenone, Mitochinone, Q10 coenzyme, vitamin E and for polyphenol components [1,35,36,37,38,39,40,41]. The administration of such compounds attenuated the consequences of the disorder at the tissue level [11,37] thus exerting protective effects against the associated degeneration.

To specify the molecular mechanism underlying the FA, in the present study use was made of rodent models carrying mutations responsible for the corresponding neurodegeneration on which the effects were tested of putative protective approaches. In particular, the *Fxntm1Mkn* mice, also called the M12 line [42], which carries the mentioned Frataxin gene mutation was chosen as a rodent model of the disorder. M12 mice should be considered as functional Frataxin KO mice as they display the histological and functional features of the FA and, in this study, they have been used for investigating the possibility of devising potentially protective approaches, based on nutraceutical, mostly polyphenol, administration. However, since the M12 mutation was itself lethal in homozygosis, in order to develop a suitable transgenic line for phenotype analysis, these mice were crossed into the Fxntm1MknTg(FXN^--^)YG8Pook/J line, which generated the M02 mice, carrying at the same time the human Frataxin (Pook) gene in emizygosis, which implied the occurrence of a variable number of supernumerary GAA triplets [43] within its first intron. This led the arising double mutant mice to reach an about 30–40% level of Frataxin biological activity, so as to survive also in homozygosis, which was crucial for the mutant line to develop the full phenotype of the disorder, against which the protective effects of pharmacological treatments, mostly based on polyphenol administration, were evaluated.

Indeed, in previous studies, evidence was provided that these compounds counteract reactive oxygen/nitrogen species generation, while inhibiting the production of pro-inflammatory cytokines and chemokines in the central nervous system [44,45,46,47,48]. Therefore, once the above-mentioned transgenic lines were generated, and the corresponding phenotypes analyzed, the present study was finalized at devising therapeutic approaches in which the obtained mutant mice were treated with antioxidants as epigallocathechin gallate (EGCG), a most abundant polyphenol (Poly) of the green tea [49,50,51], in order to counteract the oxidative stress-associated phenotype [52,53,54]. Upon completion of the treatment, the obtained mutant mice and their control littermates were analyzed both morphologically and morphometrically at the age of six months, when their neural phenotype was analyzed through cell type-specific markers, focusing on constitutive nerve cells components as β-tubulin, but also on neuronal surface adhesive proteins provided with high mobility within the plane of the membrane and involved in neurite growth control as those which belong to the Contactin family, in particular, the GPI-anchored Contactin 1 axonal adhesive glycoprotein, known to play a relevant role in neural developmental control [55]. At the same time, given its potential role in neuroprotection [56,57] the profile of the glial lineage was also evaluated through the expression of the Glial Fibrillary Acidic Protein (GFAP).

Altogether, the chosen approach allowed evaluating the relationships between neurodevelopmental markers expression and neurodegeneration events, with a special concern on the significance in such interaction of polyphenol components administration and of adhesive morphoregulatory proteins expression. In turn, this provided evidence that the approach based on polyphenol components administration was able to counteract the neurodevelopmental delay.

## 2. Results

### 2.1. Rodent Models for the Friedreich Ataxia: Cerebellar and Spinal Cord Phenotypes and Effects of Antioxidant Treatment

To reproduce the tissue phenotype triggered by Frataxin gene mutation [22,27,58], the M12 mice, carrying the mentioned FXN^--^ genotype, were analyzed in comparison to wild type (WT) littermates. In these mutants, the consequences were explored on the neural phenotype of the treatment with the EpiGalloCatechinGallate (EGCG) polyphenol for which two different protocols were alternatively applied to pregnant mothers as well as to their offsprings until the age of six months; in such mice, an EGCG concentrations in the food of 0.0185 mg/g (PolyL) or a ten-fold higher input (0.185 mg/g) (PolyH) were used. Given the predominant interest in axonal growth, the tissue phenotypes were then analyzed in both the cerebellum and the spinal cord of mutant mice versus WT littermates.

#### 2.1.1. Effects of Frataxin Gene Mutation on Cerebellar Development

**Figure 1:**
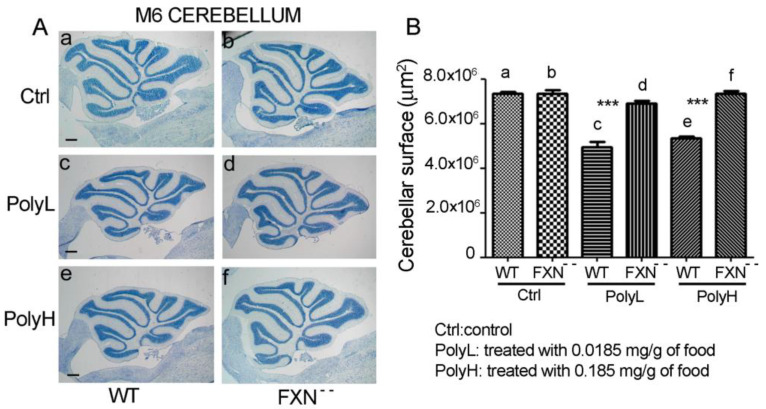
(**A**): Cerebellar phenotype of six-month-old wild type (WT) mice (a,c,e) and of littermates carrying the Frataxin gene mutation in homozygosis (FXN^--^) (b,d,f) in either control conditions (Ctrl, A (a,b)) or in the presence of EpiGalloCatechinGallate (EGCG) administration under low (PolyL: 0.0185 mg/g, c,d), or high (PolyH; 0.185 mg/g, e,f) dosage conditions. For each measure, 5 animals were used arising from either WT and FXN^--^ genotypes. Scale bar: 500 µm. (**B**): Results of the morphometric analysis measuring the surface of the whole cerebellar sections from wild type mice (WT a,c,e) and from littermates carrying the Frataxin gene mutation in homozygosis (FXN^--^ b,d,f) in either control conditions (Ctrl) or upon dietary EGCG administration by using the mentioned low (PolyL) or high (PolyH), dosage protocols. The results underwent statistical evaluation by using a one-way ANOVA test. A *p*-value < 0.0001, indicated by ***, was considered statistically significant.

In Figure 1, the phenotype of toluidine blue-stained cerebellar sections from either WT and FA-mutant mice is described, based on morphological (Figure 1A) and morphometric (Figure 1B) analyses.

In Figure 1A(a,b),B(a,b), sections from six-month-old WT and FXN^--^ mutant mice are compared. This did not reveal significant differences between the genotypes in terms of the overall tissue size, thus indicating that, in the chosen conditions and developmental stage, the mutation did not affect cerebellar ontogenesis.

We then wanted to verify whether changes in the oxidative metabolism in mutant versus wild type mice could contribute to their phenotype and for this the consequences were evaluated of polyphenol administration. In particular, the effects were measured on the cerebellar size of the treatment with EGCG, which was added to the food of WT and FXN^--^ mutant mice by using the mentioned low (PolyL: 0.0185 mg/g of food) or high (PolyH: 0.185 mg/g of food) concentration protocols. Polyphenol administration to WT mice resulted into a significant effect on the cerebellar size compared to untreated controls (Ctrl) (Figure 1A,B, compare a,c,e); in particular a 29% decrease (*p* < 0.0001) in the cerebellar section surface was observed upon PolyL treatment (a,c), while a 26% effect (*p* < 0.0001) was demonstrated when the PolyH protocol was applied (a,e). On the other hand, the same treatment, applied to Frataxin mutant (FXN^--^) mice, was essentially devoid of any significant effect as, in such conditions, the size of polyphenol-treated cerebella was only slightly reduced compared to one of untreated controls (Figure 1A,B, compare b to d and f).

Altogether, these data indicated the ability of polyphenol administration, under both the low and the high dosage protocols, to induce a developmental delay in WT mice. However, such a phenotype was lost in mice carrying the Frataxin gene mutation, in which very minor or no effects on cerebellar size could be observed. Therefore, while EGCG administration exerted inhibitory effects on the neural development of WT mice, no such an effect could be demonstrated in the case of the Frataxin-mutant (FXN^--^) mice, thus indicating that the negative EGCG effects observed on cerebellar neurogenesis of WT mice were in fact efficiently counteracted by the Frataxin gene mutation and therefore that, in the FXN^--^ mice, Frataxin gene downregulation resulted in protective effects against the neural developmental delay, which occurred as a consequence of polyphenol administration. As for the underlying mechanism, it could be supposed that, in FXN^--^ mutant mice cerebellum, Frataxin downregulation counteracted the oxidative damage resulting from the polyphenol treatment, so as to promote developmental events (precursor proliferation and differentiation) and therefore to restore neurogenesis.

To further explore the consequences of the mutation on neurodevelopmental events, the expression of neuronal and of glial lineages molecular components were also evaluated through morphological and morphometric analyses on immunostained cerebellar sections, in which antibodies directed towards cell type-specific markers were used. In particular, as for the neuronal lineage, immunohistochemical studies were performed by using antibodies directed against β-tubulin, while GFAP was taken as a glial marker. In addition, the profile of the Contactin 1 axonal adhesive glycoprotein was explored to derive concomitant information on the behavior of neuronal and oligodendrocyte components.

**Figure 2:**
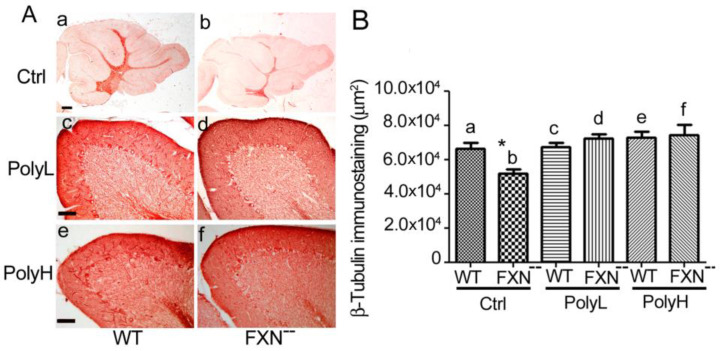
(**A**). β-tubulin expression in the cerebellum from six-month-old WT (a,c,e) and FXN^--^ mutant (b,d,f) mice. In both genotypes, vermal sections are shown in either control mice, i.e., in the absence of any treatment (Ctrl a,b), or in mice undergoing treatment with low dosage (PolyL: 0.0185 mg/g of food, c,d) or high dosage (PolyH; 0.185 mg/g of food, e,f) EGCG administration. In a,b reduced β-tubulin immunostaining indicated impaired neurogenesis in FXN^--^ mice, which underwent recovery upon polyphenol treatment under the low (PolyL, c,d) or the high (PolyH, e,f) protocols, see also the results of the morphometric analysis in Figure 2B. Scale bars: a = 500 µm; c, e = 50 µm. (**B**): The results are reported of the morphometric analysis (pixels/field) of the immunostainings shown in (**A**). A *p*-value = 0.01, indicated by *, was considered statistically significant. In FXN^--^ mice EGCG treatment under both the low (PolyL, c,d) and the high (PolyH, e,f) dosage protocols resulted into a recovery of neuronal marker expression compared to untreated controls (Ctrl) (compare d, f to b), indicating increased neuronal commitment.

As shown in Figure 2A(a,b) (see also Figure 2B(a,b)), a significant reduction (22%, *p* = 0.01) of β-tubulin immunostaining was observed all over the folium of Frataxin mutant mice (FXN^--^) compared to WT littermates in control (Ctrl) conditions, i.e., in the absence of EGCG administration, thus confirming a tendency to reduced neurogenesis in the cerebella of mice carrying the Frataxin gene mutation.

In terms of the underlying mechanism, this phenotype likely reflected the occurrence of oxidative damage in the mutant mice cerebellum and, to verify this possibility, the effects were explored on the above phenotypic trait of EGCG administration under both the mentioned low (PolyL, 2A(c,d)) and high (PolyH, Figure 2A(e,f)) dosage paradigms. As confirmed by morphometric analysis in Figure 2B, in the presence of either low (c,d) or high (e,f) conditions of EGCG administration, no significant differences in terms of β-tubulin expression could be observed in Frataxin mutant versus WT mice. However, when compared to control conditions (Ctrl), i.e., in the absence of polyphenol administration, an average 39% increase of β-tubulin expression (*; *p* = 0.01) was demonstrated in FXN^--^ mice upon EGCG treatment in both dosage (PolyL and PolyH) conditions (in Figure 2B compare d and f to b). These results indicate a positive effect of polyphenol administration on the neuronal phenotype in FXN^--^ mutant mice, and then that EGCG treatment was able to counteract the delay in cerebellar neurogenesis, which resulted from Frataxin gene mutation. Therefore, in the postnatal cerebellar cortex, polyphenol administration exerted positive and protective effects on neuronal commitment within a critical developmental stage (at the sixth postnatal month) so as to result in the recovery of the neuronal phenotype in the conditions of delayed neural development, as those, which occur in FXN^--^ mice.

#### 2.1.2. Effects of Frataxin Gene Mutation on the Spinal Cord Development

**Figure 3:**
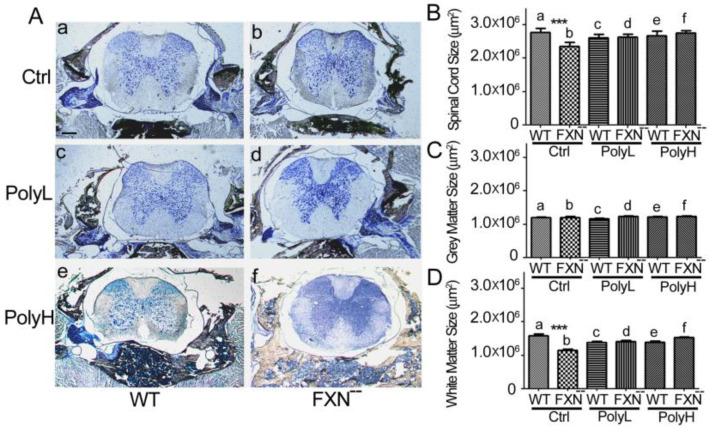
(**A**): Cervical spinal cord phenotype of 6 months-old mice in the absence (Ctrl, a,b) or in the presence of either low (PolyL, 0.0185 mg/gram of food, c,d) or, respectively, high (PolyH, 0.185 mg/gram of food, e,f) EGCG concentrations. The phenotype is reported of toluidine blue-stained spinal cord sections from either WT (a,c,e) and FXN^--^ mutant (b,d,f) mice. (**B**–**D**): Results of the morphometric analysis of the spinal cord size in the conditions shown in (**A**). (**B**)–**D** report on the analysis of sections from the whole spinal cord (**B**), or from its grey (**C**) and white (**D**) matters, respectively. An effect of the mutation in terms of the corresponding section area surfaces was demonstrated on the whole spinal cord (15.5% decrease, (**B**) a,b) as well as on the white matter (28% decrease, **D** a,b) of FXN^--^ mutant mice versus WT littermates, consistent with the occurrence of fibers atrophy. This phenotype could be efficiently counteracted by low (PolyL: (**B**), **D** c,d) as well as high (PolyH: (**B**), **D** e,f) conditions of EGCG administration in the food. On the other hand, hardly detecF effects were observed in the grey matter (**C**), confirming that the observed overall spinal cord phenotype should be mostly attributed to an effect on fibers growth rather than on perikarya. A *p*-value < 0.0001, indicated by ***, was considered statistically significant. Scale bar: 200 µm.

We then wanted to verify the effects of Frataxin gene mutation and of polyphenol administration on regions provided with longer axon tracts as the spinal cord and for this, the development of corresponding spinal segments from either WT and FXN^--^ mice were compared with the above-mentioned protocols (Ctrl, PolyL and PolyH) of EGCG treatment, with a special concern on regions bearing long range axon tracts, as the dorsal funiculus and the spinothalamic/spinocerebellar pathways. The focus was on either the whole spinal cord or, alternatively, on its white or grey matters (Figure 3A–D).

**Figure 4:**
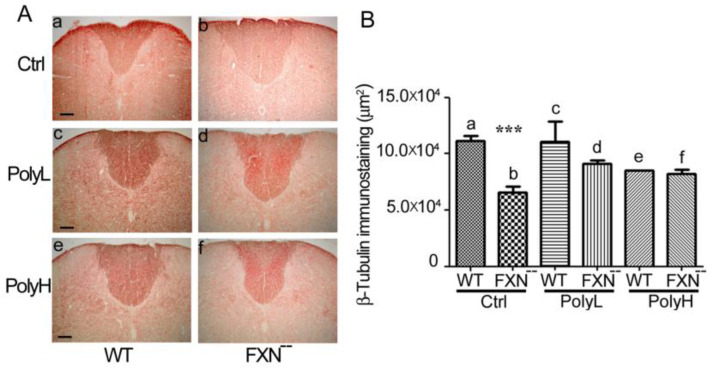
(**A**): β-tubulin expression in the dorsal funiculus of the spinal cord from WT and FXN^--^ mutant mice in control conditions (Ctrl, a,b) as well as in the presence of low (Poly L, 0.0185 mg/gram of food, c,d) or high (Poly H, 0.185 mg/gram of food: e,f) EGCG dosages. The significant β-tubulin downregulation, observed as a consequence of the Frataxin gene mutation (a,b), was efficiently counteracted upon EGCG administration under either the low (PolyL; 0.0185 mg/g) (c,d) or the high (PolyH; 0.185 mg/g) (e,f) concentration conditions. In these last case, very minor, non-significant differences in terms of β-tubulin expression could be observed. (**B**): Morphometric analysis of β-tubulin expression in the spinal cord sections shown in (**A**), reported in control conditions (Ctrl, a,b) and in conditions of low (PolyL: c,d) or high (PolyH: e,f) dietary EGCG administration. (**A**) 45% downregulation was observed in the dorsal funiculus as a consequence of FXN^--^ mutation (a,b) when a statistically significant *p*-value <0.0001, indicated by ***, was demonstrated. On the other hand, no statistically-relevant differences between the genotypes were observed in polyphenol-treated (either PolyL or PolyH) mice (c,d; e,f). Scale bar: 100 µm.

Next, we wanted to verify in the spinal cord the effects of polyphenol administration on the arising phenotype at the cellular level through the expression of cell type-specific markers. Again, the neuronal lineage development was examined by using β-tubulin as a marker, which allowed us to follow the changes in the dorsal funiculus (see Figure 4).

As shown in Figure 4A(a,b) and as confirmed by morphometric analysis in Figure 4B(a,b), in the dorsal funiculus a significant 45% (*p* < 0.05) reduction of the β-tubulin levels was revealed in FXN^--^ mice compared to WT littermates in control (Ctrl) conditions, i.e., in the absence of EGCG treatment, which was indicative of reduced development of long-range axon tracts in this region. However, this phenotypic trait was sharply counteracted upon polyphenol administration, as demonstrated by using the mentioned Low (PolyL, Figure 4A(c,d)) or High (PolyH, Figure 4A(e,f)) concentration protocols (see Figure 4B for the corresponding morphometric analysis). Indeed, mostly in this last condition, comparable β-tubulin expression levels were found in FXN^--^ mutant mice versus WT littermates. Altogether, these data indicated that, in FA spinal cord, treatment with polyphenols like EGCG exerted protective effects against the neurodevelopmental delay resulting from the Frataxin gene mutation.

**Figure 5:**
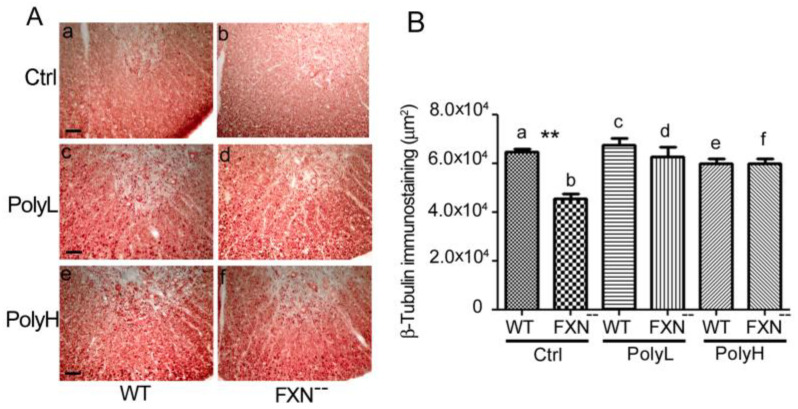
(**A**): β-tubulin expression in the ventral horn of the spinal cord from WT and FXN^--^ mutant mice in control conditions (Ctrl, a,b) or in the presence of low (PolyL: 0.0185 mg/g, c,d) or high (PolyH 0.185 mg/g, e,f) EGCG dosages in the diet. Scale bar: 50 µm. (**B**): Morphometric analysis of β-tubulin expression in the spinal cord sections shown in (**A**), reported in control conditions (Ctrl) and in conditions of low (PolyL) and high (PolyH) EGCG dietary administration. The significant reduction of β-tubulin expression observed as a consequence of the Frataxin gene mutation (18.55% *p* = 0.005) (a,b) was efficiently counteracted by EGCG treatment upon either low, PolyL (c,d), or high, PolyH (e,f) conditions. A *p*-value of 0.001, indicated by **, was considered statistically significant.

To support these effects, β-tubulin expression was also explored in the ventral horn of the spinal cord, in which the expression of such neuronal marker, shown in Figure 5, was consistent with the one observed in Figure 4, indicating a significant downregulation in FXN^--^ mutant compared to WT mice in control conditions (Figure 5A(a,b)), thus confirming in this region the neurodevelopmental delay observed in the dorsal funiculus.

Indeed, the morphometric analysis revealed in the ventral horn an 18.5% reduction of β-tubulin expression (*p* = 0.005). Similarly to the situation observed in the dorsal funiculus, no significant differences between the genotypes in terms of β-tubulin expression could be demonstrated in the presence of EGCG treatment under either the low (PolyL, Figure 5A(c,d)) or the high (PolyH, Figure 5A(e,f)) concentration protocols (see also the results of the morphometric analysis reported in Figure 5B), thus confirming that antioxidant treatment of FXN^--^ mutant mice efficiently counteracted the developmental delay occurring along the neuronal lineage.

#### 2.1.3. Contactin 1 Expression Profile

Given its significance in developmental control, the expression profile of the Contactin 1 axonal glycoprotein (CNTN1) was also explored in the spinal cord by focusing on both the dorsal funiculus and the dorsal horn. As for the former, in WT and FXN^--^ mutant mice, Contactin 1 expression was recorded both in control (Ctrl) conditions and in the presence of low (PolyL) or high (PolyH) dosage of EGCG administration.

**Figure 6:**
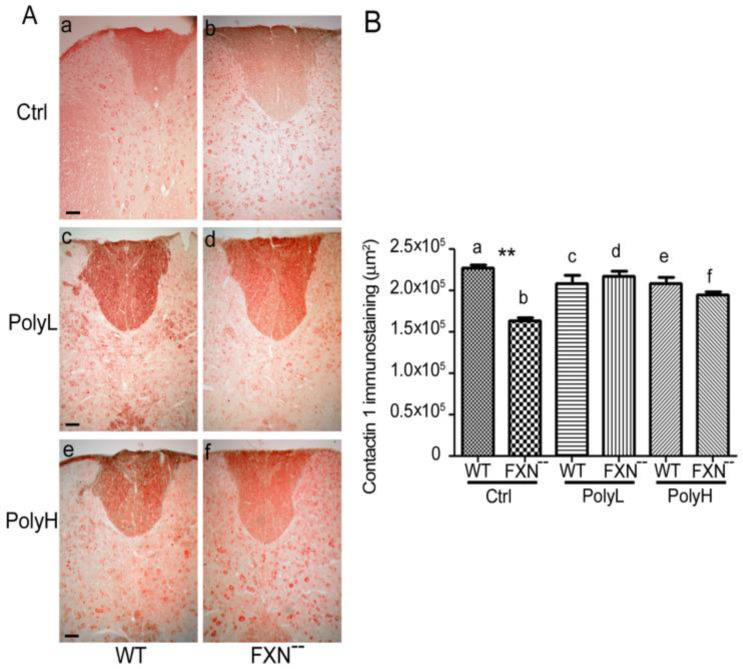
(**A**): Expression of Contactin 1 in the dorsal funiculus from WT (a,c,e) and FXN^--^ mutant (b,d,f) mice in the absence (Ctrl, a,b) or in the presence of polyphenol (EGCG) administration under the low (PolyL; 0.0185 mg/g, c,d) or the high (PolyH; 0.185 mg/g, e,f) concentration protocols. Scale bar: 100 µm. (**B**): Results of the morphometric analysis of Contactin 1 immunostaining in the conditions reported in (**A**), expressed as pixels/field. Note the significant reduction of Contactin 1 expression in FXN^--^ mutant mice in control conditions (a,b) and its recovery in the presence of EGCG polyphenol administration (both PolyL, c,d and PolyH, e,f concentration protocols). A *p*-value of 0.001, indicated by **, was considered statistically significant.

As shown in Figure 6A(a,b), a 30% downregulation of CNTN1 expression (**; *p* = 0.001) was observed in the dorsal funiculus of the FXN^--^ compared to WT mice, consistent with reduced neurogenesis occurring as a consequence of Frataxin gene mutation (see also the results of the morphometric analysis in Figure 6B). On the other hand, FXN^--^ mice undergoing administration with low (PolyL, c,d) or high (PolyH, e,f) EGCG concentrations underwent a significant recovery of CNTN1 expression in the dorsal funiculus (Figure 6A, compare c with d and e with f), further supporting the protective effects of EGCG polyphenols administration against the neurodevelopmental delay resulting from FXN^--^ gene mutation (compare also with Figure 6B).

The profile of the Contactin 1 expression was also explored in the dorsal horn of FXN^--^ versus WT mice in the presence or in the absence of EGCG administration.

**Figure 7:**
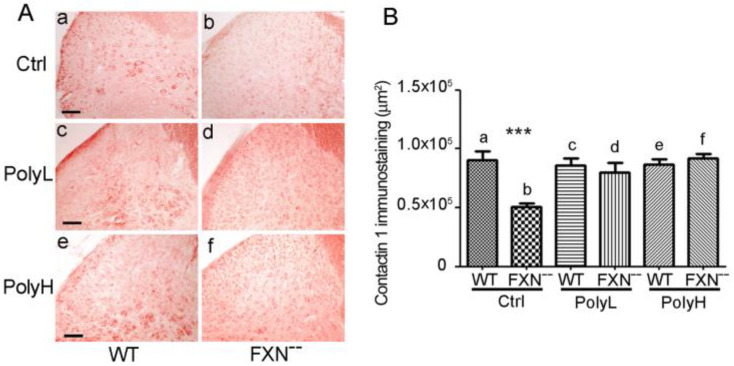
(**A**): Expression of Contactin 1 in the dorsal horn of the spinal cord from either WT (a,c,e) or FXN^--^ mutant (b,d,f) mice in control conditions (Ctrl) (a,b) or in mice undergoing low (PolyL; 0.0185 mg/g, c,d) or high (PolyH; 0.185 mg/g, e,f) dosages of EGCG polyphenol administration in the diet. Scale bar: 50 µm. (**B**): Morphometric analysis of Contactin 1 immunostaining in the conditions reported in A, expressed as pixels/field. Note the significant reduction of Contactin 1 expression in FXN^--^ mutant mice in control conditions (43%) and its recovery in the presence of polyphenol administration under low (PolyL, c,d) or high (PolyH, e,f) concentration conditions. A *p*-value < 0.0001, indicated by ***, was considered statistically significant.

In Figure 7, the CNTN1 expression profile is reported in 6 months-old mice cervical spinal cord dorsal horn. When coronal sections from FXN^--^ mice and WT littermates were compared in control (Ctrl) conditions, i.e., in the absence of EGCG administration, reduced values of Contactin 1 expression were observed in FXN^--^ mutant mice in which a 43% value (*p* < 0.0001) was observed compared to WT littermates (Figure 7A, compare a with b), which supported delayed neural development in mutant mice. In the presence of EGCG administration by using either the Low (PolyL, c,d) or the High (PolyH, e,f) concentration protocols, a recovery in Contactin 1 expression was demonstrated in FXN^--^ mice compared to controls (Figure 7A, compare c with d and e with f). In Figure 7B the results of the morphometric analysis of the Contactin 1 expression levels in the different conditions are reported. The data confirmed the significant reduction of Contactin 1 expression in FXN^--^ mutant mice compared to controls (57%, Figure 7B(a,b)), supporting the delayed neurogenesis in the former, as well as the recovery upon both the chosen (PolyL and PolyH) EGCG treatment conditions (Figure 7B(c,d),(e,f)).

Altogether, the data indicated that both in the dorsal funiculus and in the dorsal horn of the spinal cord a similar trend was observed for Contactin 1 expression, its downregulation in the presence of Frataxin mutation undergoing recovery upon polyphenol administration.

Therefore, as far as the neuronal phenotype is concerned, the above data demonstrated that the latter was generally counteracted as a consequence of the Frataxin gene mutation while undergoing recovery upon EGCG administration.

### 2.2. Glial Cells Phenotype

Next, to verify whether changes in the glial lineage could also contribute to the phenotype of FXN^--^ mice, the expression of the GFAP [59] was explored in FXN^--^ mice in comparison to wild type littermates.

**Figure 8:**
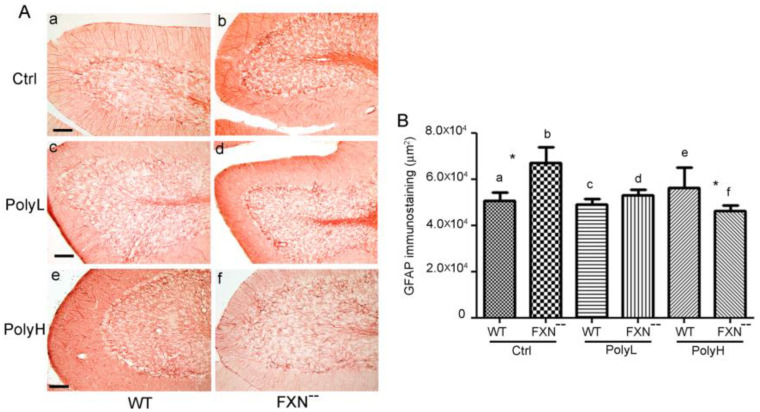
(**A**): Expression of the Glial Fibrillary Acidic Protein (GFAP) in the cerebellar folium of WT and FXN^--^ mutant mice at the 6th month. Vermal sections from FXN^--^ mice displayed a significant increase of GFAP expression compared to WT littermates in the absence of any treatment (Ctrl, a,b). On the other hand, minor differences between the genotypes were observed in the presence of treatments with either low (PolyL; 0.0185 mg/g, c,d) or, respectively, high (PolyH; 0.185 mg/g, e,f) EGCG concentrations in the diet. In this last case, a downregulation of GFAP expression was even observed. Scale bar: 50 µm. (**B**): Morphometric analysis of Glial Fibrillary Acidic Protein (GFAP) expression in the folium of WT and FXN^--^ mice reported in (**A**). A significant (*) increase in GFAP expression (32%, *p* = 0.038) was observed in FXN^--^ compared WT mice, while minor differences among the genotypes were found in the presence of low (PolyL; 0.0185 mg/g, c,d) with even a decrease in the presence of high (PolyH; 0.185 mg/g, e,f) dietary EGCG administration.

As shown in Figure 8A(a,b), in developing cerebellar cortex GFAP expression was significantly upregulated in FXN^--^ mutant mice compared to WT littermates, indicating a positive effect of the mutation on astrocyte development, which accounted to a 32% value (*p* = 0.038), as supported by morphometric analysis (Figure 8B(a,b)). Therefore, in Frataxin mutant mice, the glial phenotype was promoted at the same time as the neuronal damage and, like for the latter, the differences in the glial phenotype were counteracted upon EGCG administration under the mentioned low concentration protocol (PolyL) in which a faintly significant (*p* = 0.48) 7% increase was still observed (Figure 8A(c,d)). On the other hand, in the case of higher dietary polyphenol input (PolyH) (Figure 8A(e,f)) an 18% GFAP downregulation was rather demonstrated (*p* = 0.045). Overall, these data indicated inhibitory polyphenol effects on the glial phenotype in such conditions (see the results of the morphometrtic analysis in Figure 8B). The above findings justified the use of GFAP as a marker in order to follow the evolution of the glial phenotype in the presence of the neural damage arising from Frataxin gene mutation and confirmed that, in the cerebellar cortex, glial cells generation underwent an upregulation potentially reflecting the occurrence of a repair process. However, these effects were counteracted upon EGCG administration.

**Figure 9:**
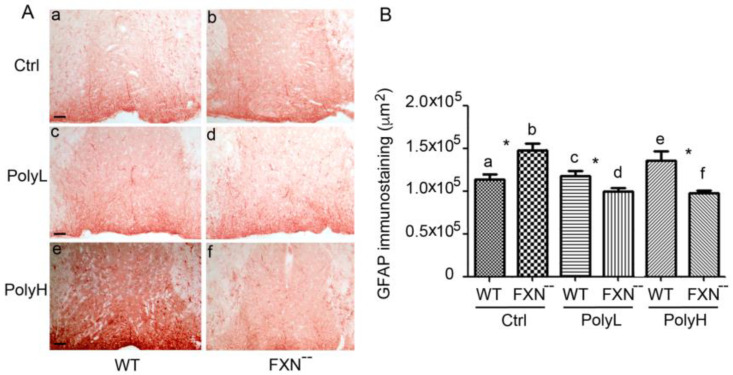
(**A**): Expression of the Glial Fibrillary Acidic Protein (GFAP) in the dorsal funiculus of the spinal cord from 6 month-old wild types (WT) and Frataxin mutant (FXN^--^) mice. Spinal cord coronal sections labeled by GFAP antibodies, demonstrate an increase of this glial marker in the dorsal funiculus of mutant mice in control conditions (Ctrl), which indicated the occurrence of reactive gliosis therein, reflecting protective effects against the oxidative damage. On the other hand, in the presence of low dosages (PolyL; 0.0185 mg/g, c,d) and mostly high dosages (PolyH; 0.185 mg/g, e,f) of EGCG treatment, GFAP expression was significantly downregulated. Scale bar: 50 µm. (**B**): The bar graphs report on the morphometric evaluation of the GFAP labeling shown in (**A**), expressed as the pixel per field. The GFAP levels were found to be increased by a 22% value in FXN^--^ mice compared to WT in control conditions with a statistically significant *p*-value of 0.01, indicated by * (a,b), while a significant downregulation was observed upon EGCG treatment, under both concentrations used (c,d and e,f).

The effects on the glial lineage were also evaluated, through the GFAP expression in the spinal cord in FXN^--^ mice versus WT littermates, which was explored in the presence of EGCG-treatment (PolyL and PolyH protocols) versus control (Ctrl) conditions. As shown in Figure 9A(a,b), upregulation of GFAP expression was demonstrated in Frataxin-mutant mice, which accounted for a 22% value, *p* = 0.01 (see morphometric evaluation in Figure 9B).

Such an effect was counteracted upon EGCG administration under the described Low (PolyL, Figure 9A(c,d)) and mostly High (PolyH, Figure 9A(e,f)) dosage protocols (see the results of the morphometric analysis reported in Figure 9B), thus reproducing in the spinal cord the effects observed in the cerebellar cortex.

## 3. Discussion

In this study, rodent models of the inherited neurological disorder known as the Friedreich Ataxia (FA) [1,3,8,13,22,60] have been used to address the significance in the corresponding phenotype of the profiles of neuronal and glial cell lineages, with a specific concern on the underlying cell-type-specific molecular substrates. At the same time, the protective effects have been evaluated of specific treatments based on antioxidants/nutraceuticals administration, in particular of polyphenol derivation.

Upon pathogenetic criteria, FA has to be considered as a mitochondrial disorder, which implies mutations within the gene encoding the Frataxin protein [24]. Such a mutation was found to affect axon tract development so that FA may be also classified among the disorders of the axonal growth and of neuronal differentiation [61,62]. Furthermore, in the present study, a phenotype was also demonstrated to occur in the course of the disorder along the glial lineage, resulting in its upregulation, potentially bearing the significance of reactive gliosis, indicative of a concomitant neuroinflammation process, known to be associated with neurorepair mechanisms [56,57].

As for the FA phenotypes, the collected data focused on the cerebellar cortex and on the spinal cord, which bears high-density long-range axon tracts. The interest of this choice was twofold: (i) to elucidate the molecular and cellular substrates of the disorder and (ii) to devise potential therapeutic approaches.

Indeed, FA has been typically considered as a neurodegenerative disorder [3,11,12,22], depending upon an imbalance of the oxidative metabolism [22,63], whose consequences at the tissue level were found to primarily concern the spinal cord in either the posterior funiculus or the spinothalamic and spinocerebellar pathways [2,7,8,11,12,28,34,64], which bear a relevant significance in modulating afferent information as well as motor function. This resulted in a complex deficit of sensory functions and of deep reflexes, in turn leading to the observed ataxic phenotype, typical of the disorder [9,16,31,32].

As far as the cell-type specificity of the FA phenotype at the tissue level, our data indicated that both neuronal and glial lineages were affected, although in different directions: the neuronal lineage was primarily concerned, as demonstrated through the consistent early β-tubulin downregulation while a glial upregulation was rather demonstrated through the GFAP glial marker levels. In turn, this bore the significance of reactive gliosis, known to participate in distinct neurorepair [56,57,65,66,67], as well as neuroinflammation [57,68,69] conditions. As indicated, although spanning the same developmental window, the neuronal and the glial phenotypes underwent opposite changes, neuronal downregulation being associated with positive effects on the glial lineage. However, these opposite effects were sustained by similar pathogenetic mechanisms as they were both counteracted upon antioxidant (EGCG) administration [28,65], consistent with the hypothesis that FA is to be primarily considered as a disorder of the oxidative metabolism [11,12,22,41]. In addition, this is also consistent with the protective effects of antioxidant treatment against tissue damage, demonstrated in distinct neurodegenerative disorders including Parkinson disease [70,71], Alzheimer Disease [72], as well as some forms of dementia [51,73,74,75,76].

As indicated, the neuronal phenotype could be easily demonstrated through the β-tubulin expression profile. However, stronger effects were demonstrated in the case of neural adhesive glycoproteins provided with key relevant regulatory roles in neural developmental control as is typically the case for Contactin family components [77] and in particular for Contactin 1 [55,78], for which a stronger downregulation, accounting to about 50% was demonstrated in the dorsal horn. This Contactin 1 behavior could be explained based on its cell type-specificity, which, besides the neuronal lineage, also concerns the glial, in particular the oligodendrocyte lineage in which it participates to the mechanism of myelination through its effects on nodal/paranodal regions organization [55,77,78,79,80,81,82,83,84,85,86,87,88,89].

Accordingly, in agreement with the relevant developmental role of Contactins, changes in their expression profiles were suggested to sustain the pathogenesis of specific neurological disorders [90,91,92,93] and in the present study, we provide evidence about the potential involvement of Contactin 1 into the pathogenesis of the Friedreich Ataxia in which we report reduced Contactin 1 expression in both the cerebellar cortex and the spinal cord. This is in agreement with the Contactin 1 neuronal/axonal surface location, which sustains its significance in axonal growth control, and with its expression on myelinating cells. In turn, this indicates a pleyotropic function for the expression of this molecule, which, besides neuronal commitment and differentiation, also supports its relevant role in action potential generation and conduction [55,77].

In general terms, the observed profiles of Contactin 1 expression are consistent with the significance of such molecule in exerting a widespread neuro-regulatory role, in turn depends upon its ability to activate pathways provided with a general relevance in developmental control, with reference, in particular, to the one associated with Notch receptors activation [94,95]. Indeed, based on the close correlation of Contactin 1 expression with Notch pathway activation [55,96,97], downregulation of such axonal glycoprotein, we observed in the damaged nervous tissues in the FA, is expected to exert negative effects on the Notch pathway, which, based on the inhibitory role of the latter on neurogenesis and neural development [94], should result in positive consequences on neurogenesis. Therefore, these data sustain the possibility that the observed negative modulation of Contactin 1 expression and activity in the course of FA is likely provided with a relevant significance in promoting neural repair and it is, therefore, worth emphasizing that the corresponding mechanisms may be related to the one exerted by polyphenols, which also correlate with modulation of the Notch pathway activation [97,98]; indeed, on a related topic, taking into account the specific involvement of the above mechanism in neuroinflammation, we demonstrated in previous studies by using the same rodent models concomitant anti-inflammatory and anti-allergic effects, through the determination of splenic cytokines [99].

Therefore, taken together, downregulation of Contactin 1 in the FA rodent models correlates with negative effects on the Notch pathway, in turn resulting in positive effects on neurogenesis, as well as in anti-inflammatory activities and in the context of the present study, such a role was consistent with the effects generally exerted by polyphenol administration on neural repair, known to involve Notch pathway downregulation [94,98,100].

In general terms, the above considerations are consistent with the hypothesis that some neurological disorders may imply modulation of the regulatory functions exerted by Contactin family components [55,77,100,101] and in this context, the demonstration that some of these molecules may also occur in soluble form in the cerebrospinal fluid [92], may provide at the same time a powerful diagnostic tool, typically demonstrated in some forms of congenital myopathies [102].

In addition, due to the mentioned Contactins involvement in myelination [55,89,103,104], changes in their expression may also concern disorders of myelinated axons and therefore of nerve conduction velocities [105]. In this context, besides the described effects on the neuronal lineage, in the present study, specific consequences were also demonstrated on the glial lineage by using a glia-specific marker as the GFAP, whose expression underwent a significant increase in the course of the disorder, thus justifying that a glia upregulation accompanied the observed neuronal degenerative phenotype, confirming the simultaneous activation of a neurorepair mechanism [56,57,66,67,106].

Therefore, overall, a relevant aspect which arises from the present study is that the mechanisms which drive regulated expression of axonal morphoregulatory molecules may play relevant roles not only for setting the modulation of neural developmental events but also for activating signaling mechanisms involved in neural repair, in which a relevant role may be also played by polyphenols such as EGCG.

## 4. Materials and Methods

### 4.1. Transgenic Mice Lines

For the attempts of this study, mutant lines for the rodent Frataxin gene were used. The Fxntm1Mkn mice [107,108] carry a targeted mutation disrupting the Frataxin gene exon 4 through a flanking PGK-neo cassette and will be referred here as the M12 line. This mutation, which is lethal in homozygosis, was crossed into YG8R mice, carrying two tandem copies of the human FXN gene, including GAA repeats of 82 and 190 nucleotides within its first intron. This generated the double mutant Fxntm1Mkn Tg(FXN)YG8Pook/J transgenic line, which was heterozygote for the Fxntm1Mkn mutation and hemizygote for the YG8R transgene, and will be called here the M02 line. Therefore, crossing the M12 with the M02 lines allowed to generate double mutant mice carrying the Fxntm1Mkn mutation in homozygosis and the Tg(FXN)YG8Pook/J mutation in emizygosis that in the present study we will call FXN^--^. This represented a valuable animal model for the FA, in which the rodent Frataxin gene (FXN) was suppressed while its human counterpart (Pook) was downregulated as a consequence of the mentioned triplet insertions within its first intron [42]. Overall, this resulted in a 30–40% Frataxin expression level and, in this line, neural development was demonstrated to be sharply affected. All information is available at the Jackson’s lab web page (https://www.jax.org/strain/008398).

**Figure 10:**
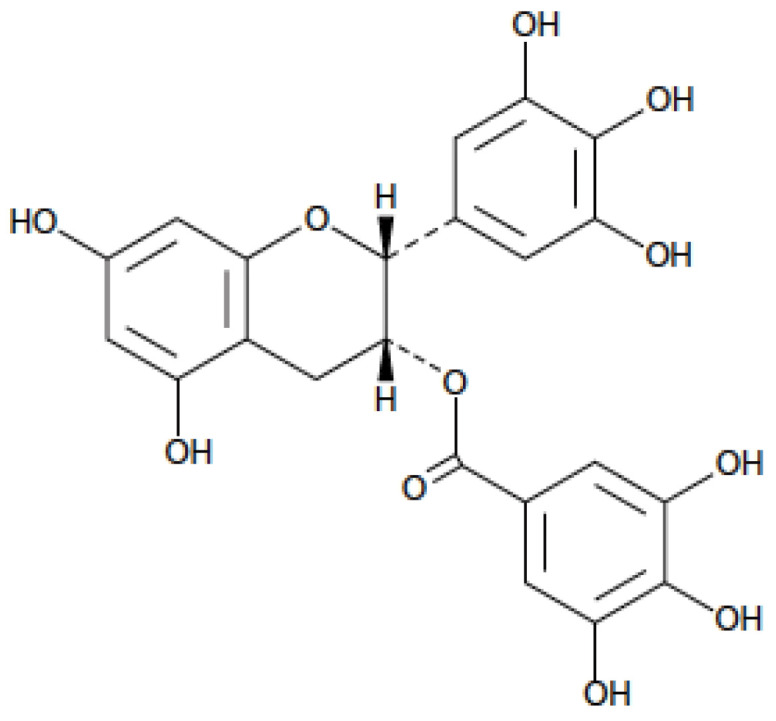
Structure of epigallocathechin gallate.

In a related part of this study, in the above mice lines, the potential protective effects of polyphenol administration against the consequences of the mutation were evaluated with reference, in particular, to the epigallo-catechin gallate (EGCG, Sigma Aldrich), which was either directly incorporated into the food of pregnant mothers or, alternatively, of their offsprings during postnatal development. In both cases, two different schedules were alternatively followed, implying EGCG concentrations in the food of 0.0185 mg/g (low concentration: PolyL) or a ten-fold higher concentration (0.185 mg/g: PolyH). In addition, a further rodent population assumed food devoid of EGCG, so as to provide an adequate negative control (Ctrl) to the whole procedure. In preliminary experiments, EGCG doses were selected using a range of concentrations from 0.0010 mg/g to 0.50 mg/g. The most appropriate doses were comprised between 0.0185 and 0.185 mg/g. The structure of EGCG is indicated in Figure 10.

### 4.2. Phenotype Analysis

Offspring phenotype analysis was carried out at the morphological and morphometric levels in six-month-old mutant mice and in their wild type littermates. For this, mice were perfused with 4% paraformaldehyde (PFA) in 0.12M phosphate buffer (PB) pH 7.4 and then the tissues of interest were dissected out and post-fixed overnight in the same fixative. Twelve µm cryostat sagittal sections were then generated and immunostainings were performed on corresponding fields of the cerebellar vermis, whose derivation along the medio-lateral axis was deduced upon comparison with adjacent structures.

A similar analysis was devised in the spinal cord, in which horizontal sections, generated from corresponding regions, were stained with either toluidine blue, or with antibodies against neuronal/glial markers and, again, a morphological/morphometric analysis was carried out.

### 4.3. Morphological and Morphometric Analysis

Tissue sections were immunohistochemically analyzed by using primary antibodies directed toward cell type-specific markers. In particular, reagents directed towards β-tubulin (mouse monoclonal, Novus Biologicals, Cambridge UK), GFAP (mouse monoclonal, Novocastra Laboratories, Newcastle, UK) and a rabbit antiserum towards Contactin 1 [101] were used. Mice were perfused with 4% paraformaldehyde in 0.12 M PB. 12 µm sections were then generated from frozen tissues and permeabilized for 30° at room temperature (RT) in PBS containing 0.5% Triton X-100, 3% BSA, 5% FCS and then incubated ON at 4 °C with primary antibodies diluted in the same solution except that 0.25% replaced 0.5% Triton X-100. Finally, after washing, sections were incubated with biotinylated secondary antibodies (1 h at RT) followed by Streptavidin Peroxidase (1 h incubation at RT) and by 3-amino-9-ethylcarbazole (AEC, 15-20′ at RT).

Immunostained sections were analyzed by using a TCS-SP8 laser-scanning microscope (Leica Microsystems, Wetzlar, Germany), through a sequential scanning procedure. Confocal images were taken at 1 μm intervals through the z-axis of the sections with 20× or 40× lenses. The acquired images were digitized, optimized by contrast enhancement functions and segmented by an interactive modality of the program. The resulting binary images were then processed by functions allowing measurement of the extent of the immunostaining, deduced by the pixel number. Histomorphometric analyses were done on selected corresponding sections by using the Image J software (Image J v. 1.52a, National Institute of Mental Health, Bethesda, Washington, MD USA) [109].

### 4.4. Statistical Analyses

To proceed to the statistical evaluation of the obtained results, all measures were performed in sections from either the cerebellum and the spinal cord from at least five animals. Twenty sections were generated for each measure and used for morphometric analysis. Mean values ± SEM were calculated and subjected to statistical analysis. For this, differences were evaluated by a one-way ANOVA test and a *p* < 0.05 value was considered statistically significant. The following variables: “Cerebellar size”, “β-tubulin”, “GFAP” and “Contactin 1” expression approached a Gaussian distribution and a linear model. A one-way factorial ANOVA model was applied in order to evaluate the effects of the polyphenol treatment on the different parameters. The results were described as least square means with 95% confidence intervals. Finally, p-values for post-hoc comparisons were adjusted according to Tukey.

### 4.5. Animal Breeding

Transgenic mice and control littermates were bred in the “Department of Basic Medical Sciences, Neurosciences and Sensory Organs”, Medical School, University of Bari, Italy and their experimental utilization conformed to the National Institute of Health Guide of the Care and use of the laboratory animals (NIH publications N. 80–23, revised 1996) and followed the Italian Ministry of Health indications (law of March 4, 2014, n. 26) under the Authorization n. 826/2017-PR of 23.10.2017. In the context of the above procedures, all efforts were made in order to minimize the number of animals used and their suffering.
